# Modification of tumour cell metabolism modulates sensitivity to Chk1 inhibitor-induced DNA damage

**DOI:** 10.1038/srep40778

**Published:** 2017-01-20

**Authors:** Andrew J. Massey

**Affiliations:** 1 Vernalis Research, Granta Park, Cambridge, CB21 6GB, UK.

## Abstract

Chk1 kinase inhibitors are currently under clinical investigation as potentiators of cytotoxic chemotherapy and demonstrate potent activity in combination with anti-metabolite drugs that increase replication stress through the inhibition of nucleotide or deoxyribonucleotide biosynthesis. Inhibiting other metabolic pathways critical for the supply of building blocks necessary to support DNA replication may lead to increased DNA damage and synergy with an inhibitor of Chk1. A screen of small molecule metabolism modulators identified combinatorial activity between a Chk1 inhibitor and chloroquine or the LDHA/LDHB inhibitor GSK 2837808A. Compounds, such as 2-deoxyglucose or 6-aminonicotinamide, that reduced the fraction of cells undergoing active replication rendered tumour cells more resistant to Chk1 inhibitor-induced DNA damage. Withdrawal of glucose or glutamine induced G1 and G2/M arrest without increasing DNA damage and reduced Chk1 expression and activation through autophosphorylation. This suggests the expression and activation of Chk1 kinase is associated with cells undergoing active DNA replication. Glutamine starvation rendered tumour cells more resistant to Chk1 inhibitor-induced DNA damage and reversal of the glutamine starvation restored the sensitivity of tumour cells to Chk1 inhibitor-induced DNA damage. Chk1 inhibitors may be a potentially useful therapeutic treatment for patients whose tumours contain a high fraction of replicating cells.

Maintaining the integrity of and faithfully copying genetic information are critical for cellular health. Failure to do so can result in persistent DNA damage leading to apoptosis or cellular senescence as well as genome instability and ultimately cancer. Decreased DNA replication fidelity through impaired fork progression, deregulated origin usage, changes to the chromatin environment or oncogene activation, and/or loss of tumour suppressor gene function increase replication stress^1^,^2^,^3^. A series of sophisticated cell cycle checkpoint and DNA repair pathways (collectively termed the DNA damage response (DDR)) have evolved to allow cells to cope with the high levels of DNA damage sustained by the genome from endogenous and environmental sources on a daily basis. ATR and Chk1 kinases, key components of the S-phase checkpoint, are critical for the cellular response to replication stress^4^,^5^,^6^. Replication fork stalling results in the generation of tracts of ssDNA as the replicative helicase continues to unwind DNA in front of the stalled DNA polymerase. Binding of ssDNA by RPA recruits ATR and its subsequent activation by TOPBP1 leads to Chk1 phosphorylation on serine 317 and serine 345^7^,^8^, and autophosphorylation on serine 296^9^. Activation of ATR and Chk1 induces cell cycle arrest (through the degradation of Cdc25 phosphatases), fork stabilisation and inhibition of cleavage by the Mus81-Eme1-Mre11 nucleases, activation of homologous recombination repair and inhibition of new origin firing. Stabilisation and protection of replication forks allows fork restart once the source of fork arrest has been removed or bypassed by DNA damage mechanisms.

Biochemical and genetic studies have demonstrated Chk1 to be essential and indispensable for the S-phase checkpoint^10^,^11^ and plays a critical role in the cellular response to replication stress. Numerous inhibitors of Chk1 have entered pre-clinical and clinical development (reviewed in refs 12 and 13). The pre-clinical and clinical development of these inhibitors has focussed on their ability to potentiate the cytotoxicity of genotoxic chemotherapy drugs (such as gemcitabine, irinotecan or cisplatin) or ionising radiation. All of these agents induce DNA damage and activate the DDR resulting in cell cycle arrest. Inhibition of Chk1 following genotoxic stress induced by these agents results in checkpoint abrogation, inhibition of DNA repair and induction of cell death particularly in cells with a defective p53 response. This approach is currently being evaluated in a range of Phase I and II clinical trials.

The increased proliferative drive of cancer cells requires a ready supply of nutrients to generate the building blocks to support cell growth and division. The metabolic properties of cancer cells are inherently different from those of normal cells^14^,^15^. These are characterised by high glucose consumption with glycolysis utilised in preference to oxidative phosphorylation to generate ATP (‘the Warburg effect’)^16^. This glycolytic switch is intrinsically linked to transformation as it is promoted by oncogenes and inhibited by tumour suppressors. In addition, cancer cells have additional metabolic changes including increased fatty acid synthesis and a high dependence on glutamine (‘glutamine addiction’)^17^. A class of drugs termed the antimetabolites have been a component of cancer therapy for decades. These drugs, which include pemetrexed, gemcitabine and hydroxyurea, generally work by inhibiting enzymes critical for nucleotide or deoxyribonucleotide biosynthesis decreasing the pool of dNTPs available for DNA synthesis thereby blocking cell proliferation and increasing replication stress. Inhibition of nucleotide and deoxyribonucleotide biosynthesis with antimetabolites activates Chk1 and the greatest potentiation of chemotherapy by Chk1 inhibitors has been observed with this class of drugs^18^. Chk1 inhibition, in combination with antimetabolite chemotherapy, results in the collapse and subsequent cleavage of stalled replication forks, increased DNA double strand breaks and cell death via apoptosis, necrosis, mitotic catastrophe or senescence.

Inhibiting other metabolic pathways critical for the supply of building blocks necessary to support DNA replication may lead to increased replication stress and synergy with an inhibitor of Chk1. Here, we evaluated the effect of numerous small molecule metabolism modulators to increase replication stress and activate the DNA damage response in combination with a novel Chk1 inhibitor.

## Results

### A screen of small molecule metabolism inhibitors identified combinatorial activity between a Chk1 inhibitor and chloroquine or GSK 2837808A

Chk1 inhibitors potentiate the activity of antimetabolite drugs that increase replication stress through the inhibition of nucleotide or deoxyribonucleotide biosynthesis. Inhibiting other metabolic pathways critical for the supply of building blocks necessary to support DNA replication may lead to increased replication stress and synergy with an inhibitor of Chk1.

**Figure 1:**
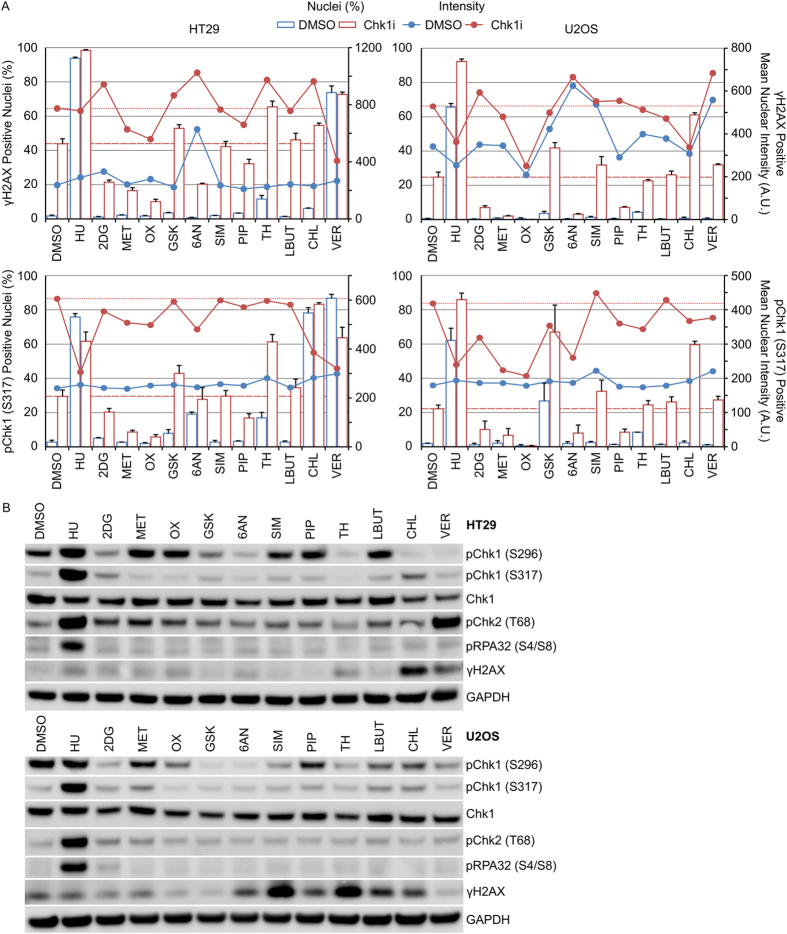
Screen of metabolism modulators to detect combinatorial activity with Chk1i. (**A**) HT29 or U2OS cells were treated with indicated combinations of metabolism modulator with either 0 or 0.4 μM Chk1i for 24 hours. The fraction of nuclei scored positive for γH2AX or pChk1 (S317) along with the mean nuclear intensity of the positive cells was determined using single cell immunofluorescent imaging (n = 4, mean ± SD). Dotted lines indicate Chk1i single-agent activity. (**B**) Cell lysates prepared from HT29 (upper) or U2OS (lower) cells treated with the indicated metabolism modulators for 24 hours were immunoblotted using the indicated antibodies (n = 1).

Treatment of cancer cells with hydroxyurea increased the fraction of cells staining positive for γH2AX and pChk1 (S317) (Fig. 1A). This correlated with increased phosphorylation of serine 296 and RPA32 on serine 4 and 8 (Fig. 1B). We screened a range of compounds capable of modulating cellular metabolism (Table 1) for their potential to increase γH2AX, a marker of DNA damage^19^ or pChk1 (S317), a marker of ATR activation, either alone or in combination with the Chk1 inhibitor V158411 (Chk1i), in HT29 and U2OS cancer cells. V158411 is a potent, selective inhibitor of Chk1 that exhibits activity as a single-agent and in combination with cytotoxic chemotherapy^20^,^21^,^22^,^23^. The response to the metabolism modulating agents was dependent on the agent and the cell line but could be broadly categorised into the following groups: (i) monotherapy increased the fraction of γH2AX and pChk1 (S317) positive cells (HU and VER); (ii) combination with Chk1i increased the fraction of γH2AX and pChk1 (S317) positive cells (GSK, CHL and TH); (iii) combination with Chk1i decreased the fraction of γH2AX and pChk1 (S317) positive cells (2DG, MET, OX, 6AN and PIP); (iv) had no effect on the fraction of γH2AX or pChk1 (S317) positive cells as monotherapy or in combination with Chk1i (SIM and LBUT) (Fig. 1A and Supplementary Table S1). As monotherapies no agents, apart from the HU control, increased pChk1 (S296), pChk1 (S317) and pRPA32 (S4/S8) (Fig. 1B and Supplementary Tables S2 and S3).

### Chloroquine or GSK 2837808A increase Chk1 inhibitor induced DNA damage

**Figure 2:**
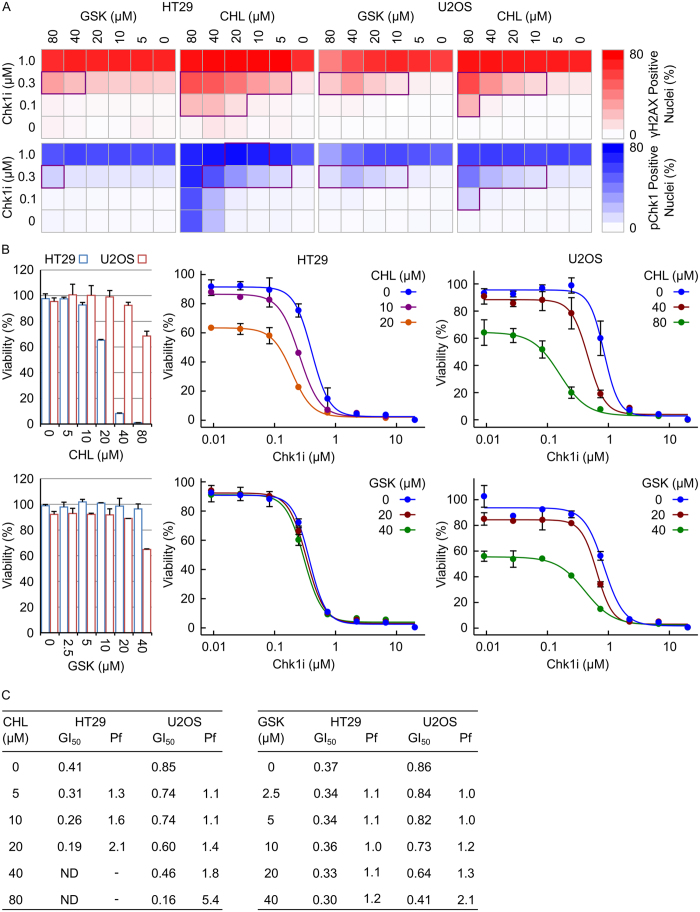
Chloroquine or GSK 2837808A increase Chk1 inhibitor induced DNA damage. (**A**) HT29 or U2OS cells were treated with a combination of Chk1i and either GSK or CHL for 24 hours. The fraction of γH2AX positive or pChk1 (S317) positive nuclei was determined by single cell immunofluorescent imaging (n = 2, mean). Combinations of the two inhibitors exhibiting synergy (as determined by a Bliss Independence CI < 0.75) are highlighted. (**B**) HT29 or U2OS cells were treated with a combination of Chk1i and either GSK or CHL for 72 hours. Cell viability was determined by SRB staining after TCA fixation (n = 3, mean ± SD). (**C**) GI_50_ values for Chk1i were calculated using XLFit software. ND, not determinable; Pf = GI_50−CHL or GSK_/GI_50+CHL or GSK_.

The observation that GSK or CHL increased Chk1i-induced DNA damage was confirmed across a range of GSK, CHL and Chk1i concentrations. Synergistic increases in γH2AX and pChk1 (S317) was observed between either GSK or CHL and Chk1i in HT29 or U2OS cells (Fig. 2A and Supplementary Fig. S1). A greater increase in Chk1i induced γH2AX and pChk1 (S317) was observed with CHL than GSK.

The ability of CHL or GSK to reduce cell viability in combination with Chk1i was assessed in HT29 and U2OS cells. CHL exhibited significantly greater single-agent activity in HT29 cells compared to U2OS cells with almost complete growth inhibition observed following 72 hour treatment with 40 or 80 μM CHL (Fig. 2B). At minimally toxic doses, CHL reduced cell viability in combination with Chk1i 2.1- and 5.4-fold in HT29 and U2OS cells respectively (Fig. 2B and C). This corresponded to increased drug synergy in the HT29 cell line (Supplementary Fig. S1). No change in cell viability was observed in HT29 cells treated with a combination of Chk1i with concentrations of GSK up to 40 μM. In comparison, 40 μM GSK reduced cell viability in combination with Chk1i by 2.1-fold in U2OS cells (Fig. 2B and C).

**Figure 3:**
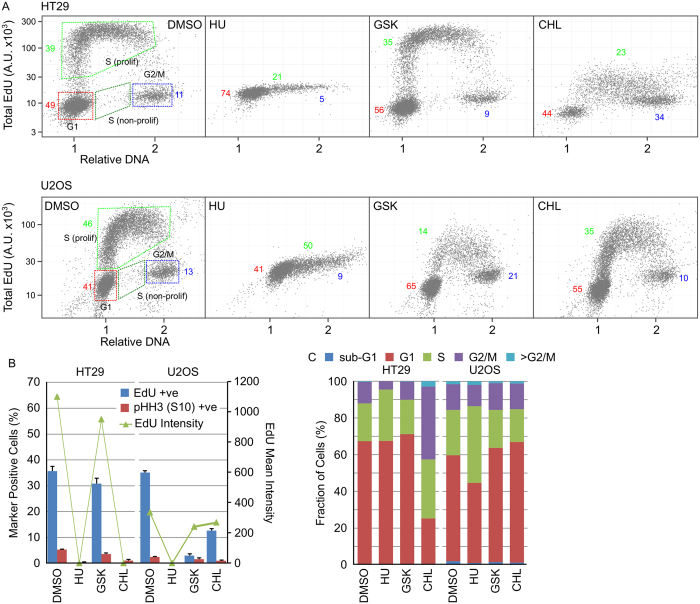
GSK and CHL inhibit active DNA replication in HT29 and U2OS cells. HT29 or U2OS cells were treated with 2.5 mM HU, 40 μM GSK or 80 μM CHL for 24 hours then EdU for a further 15 minutes. DNA content, EdU incorporation and pHH3 (S10) expression was determined using single cell immunofluorescence analysis. (**A**) Single cell plots of relative DNA content versus total nuclear EdU demonstrating the different cell cycle populations. Numbers indicate the percentage of cells in each cell cycle phase (red, G1; green, S; blue, G2/M). (**B**) Quantification of cell populations positive for EdU incorporation or pHH3 (S10) expression (n = 3, mean ± SD). (**C**) Determination of cell cycle distribution based on total nuclear DNA content (n = 3, mean).

The effect of GSK or CHL on HT29 or U2OS cell cycle distribution was determined and compared to changes induced by HU. In HT29 and U2OS cells, HU inhibited DNA synthesis resulting in G1 and S-phase arrest (Fig. 3A,B and C). GSK in U2OS cells and CHL in HT29 cells inhibited DNA synthesis (as measured by decreased EdU incorporation). In the GSK treated U2OS cells, arrest occurred in G1 and G2 phases whist in CHL treated HT29 cells, the cells arrested in G2. CHL in U2OS cells induced G1 and early S-phase arrest whilst GSK in HT29 cells did not alter the cell cycle distribution compared to the DMSO control (Fig. 3A,B and C).

### Decreased sensitivity to Chk1i is associated with a reduction in active cell proliferation

**Figure 4:**
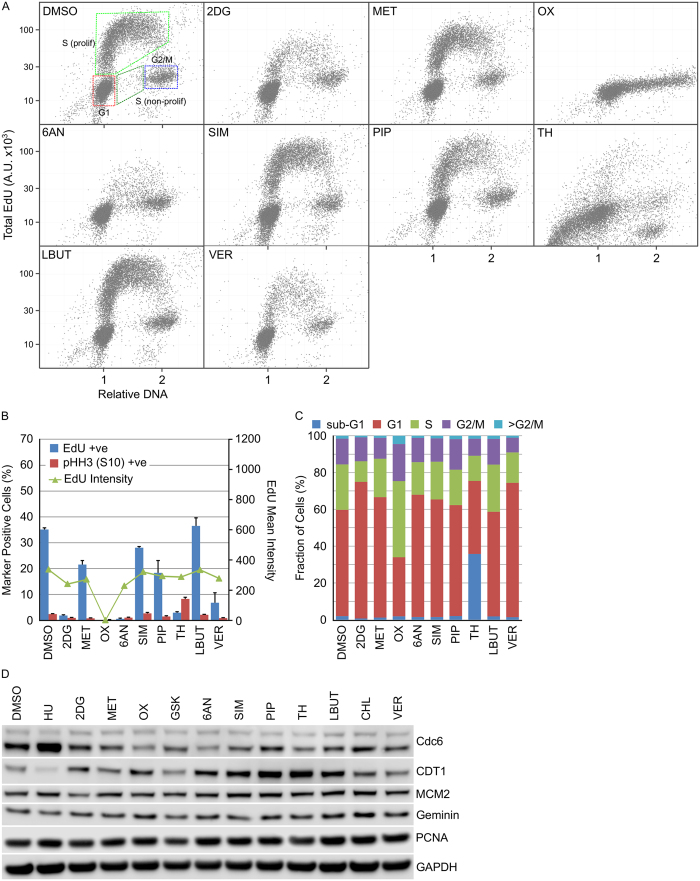
Cell cycle distribution in U2OS cells is differentially affected by metabolism modulators. U2OS cells were treated with the indicated metabolism modulators for 24 hours then EdU for a further 15 minutes. DNA content, EdU incorporation and pHH3 (S10) expression was determined using single cell immunofluorescence analysis. (**A**) Single cell plots of relative DNA content versus total nuclear EdU demonstrating the different cell cycle populations. (**B**) Quantification of cell populations positive for EdU incorporation or pHH3 (S10) expression (n = 3, mean ± SD). (**C**) Determination of cell cycle distribution based on total nuclear DNA content (n = 3, mean). (**D**) Cell lysates prepared from HT29 cells treated with the indicated metabolism modulators were immunoblotted using the indicated antibodies (n = 1).

**Figure 5:**
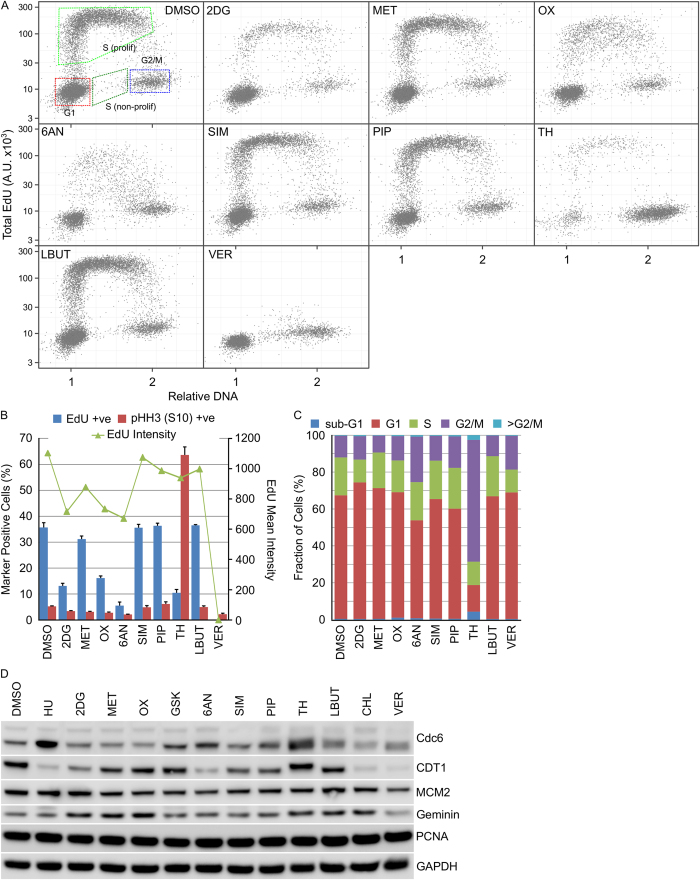
Cell cycle distribution in HT29 cells is differentially affected by metabolism modulators. HT29 cells were treated with the indicated metabolism modulators for 24 hours then EdU for a further 15 minutes. DNA content, EdU incorporation and pHH3 (S10) expression was determined using single cell immunofluorescence analysis. (**A**) Single cell plots of relative DNA content versus total nuclear EdU demonstrating the different cell cycle populations. (**B**) Quantification of cell populations positive for EdU incorporation or pHH3 (S10) expression (n = 3, mean ± SD). (**C**) Determination of cell cycle distribution based on total nuclear DNA content (n = 3, mean). (**D**) Cell lysates prepared from HT29 cells treated with the indicated metabolism modulators were immunoblotted using the indicated antibodies (n = 1).

The effect of the small-molecule metabolism modulators 2DG, MET, OX, 6AN, SIM, PIP, TH, LBUT or VER on cell cycle distribution was determined. 2DG, OX, 6AN, TH and VER all decreased the fraction of actively replicating cells as determined by the fraction of U2OS cells incorporating EdU after 24 hour compound treatment (Fig. 4A and B). In HT29 cells, a similar pattern was observed with 2DG, OX, 6AN, TH and VER reducing the fraction of EdU positive cells (Fig. 5A and B). Further analysis of this reduction in active replication identified TH induced cell death in U2OS cells (Fig. 4C) and mitotic arrest in HT29 cells (Fig. 5B and C) whilst OX induced S-phase arrest in U2OS cells (Fig. 4C). No apparent changes in cell cycle associated proteins was observed (Figs 4D and 5D).

### Expression and activation of Chk1 kinase is associated with actively proliferating cells

**Figure 6:**
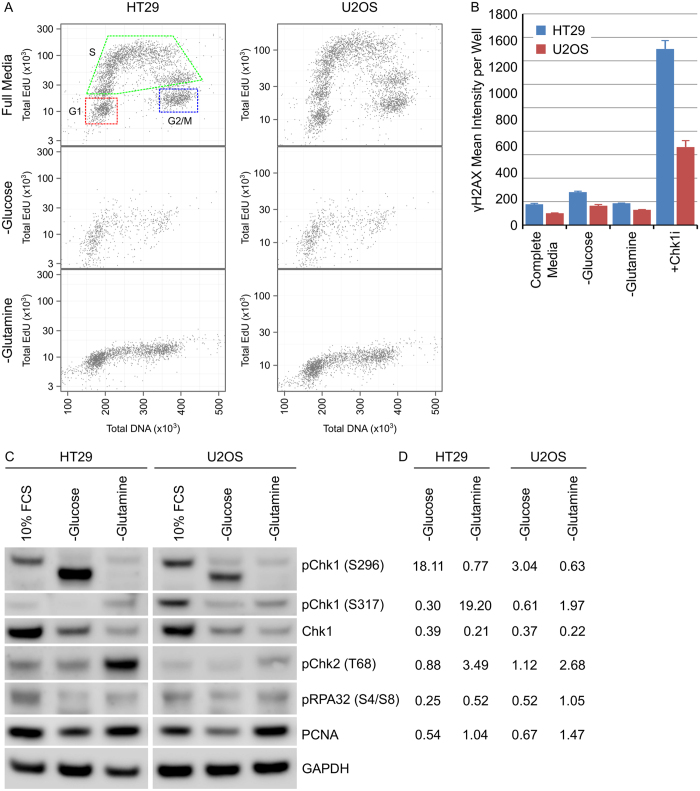
Chk1 expression and activation is associated with actively proliferating cells. (**A**) HT29 or U2OS cells were grown in complete or glutamine deficient media for 24 hours then labelled with EdU for 15 minutes. Total nuclear intensity of EdU or DNA was determined by single cell immunofluorescent imaging and plotted. (**B**) The mean γH2AX intensity per cell per well was determined using single cell immunofluorescent analysis in HT29 or U2OS cells growing in complete, glucose deficient or glutamine deficient media, or in complete media with 1 μM Chk1i for 24 hours (n = 4, mean ± SD). (**C**) HT29 or U2OS cells were grown under the indicated culture conditions for 24 hours. Cell lysates were immunoblotted using the indicated antibodies (n = 1). (**D**) Expression levels were quantified by densitometric analysis. pChk1 (S296) and pChk1 (S317) were normalised to Chk1 expression levels with all other proteins normalised to GAPDH. The fold change relative to 10% FCS was calculated.

Chk1 kinase plays a critical role in protecting cells from replication stress. Determining the cell cycle phases associated with Chk1 expression and activation is difficult as the majority of agents used to induce cell synchronisation (such as hydroxyurea or nocodazole) also induce DNA damage. Metabolic reprogramming in cancer cells increases the cellular demand for glucose and glutamine to provide the necessary building blocks for biosynthesis. Withdrawal of glutamine for 24 hours inhibited DNA synthesis resulting in G1 and G2/M cell cycle arrest (Fig. 6A) without inducing DNA damage (as measured by γH2AX expression, a marker of DNA double strand breaks, Fig. 6B) in HT29 and U2OS cancer cells. Growth in media completely depleted of glucose was detrimental to cell viability and resulted in significant cell detachment and loss after 24 hours. Cell cycle arrest induced by glutamine starvation reduced total Chk1 protein levels as well as phosphorylation on serine 296, a biomarker of Chk1 kinase activity (Fig. 6C,D and Supplementary Fig. S2). Glucose starvation also reduced Chk1 total protein levels but increased phosphorylation of a putatively truncated variant of Chk1 on serine 296. Chk1 has previously been demonstrated to be activated by cleavage during apoptosis^24^ and may reflect the protein species detected here.

### Reversal of nutrient starvation restores the sensitivity of tumour cells to Chk1 inhibitor induced DNA damage

**Figure 7:**
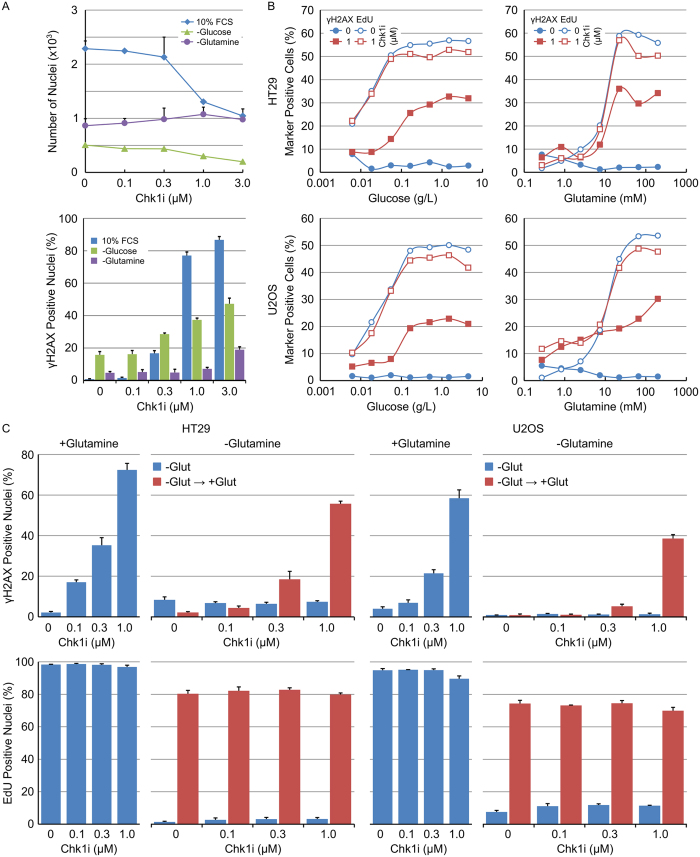
Sensitivity of tumour cells to Chk1 inhibitor induced DNA damage is restored on reversal of nutrient starvation. (**A**) HT29 cells were grown in complete, glucose deficient or glutamine deficient media in combination with the indicated concentrations of Chk1i for 24 hours. The number of nuclei (upper) or the fraction of γH2AX positive nuclei (lower) was determined by single cell immunofluorescent analysis (n = 4, mean ± SD). (**B**) HT29 or U2OS cells growing in decreasing amounts of glucose or glutamine were treated with 0.3 μM Chk1i for 24 hours. The fraction of γH2AX positive or actively proliferating (EdU positive) nuclei was determined by single cell immunofluorescent imaging (n = 4, mean ± SD). (**C**) HT29 or U2OS cells were grown in glutamine deficient media for 18 hours before being treated with the indicated concentrations of Chk1i with or without 200 mM glutamine (all in the presence of EdU) for a further 24 hours. The fraction of γH2AX positive or actively proliferating (EdU positive) nuclei was determined by single cell immunofluorescent imaging and compared to cells grown in full media (n = 4, mean ± SD).

The effect of glucose or glutamine starvation on the sensitivity of tumour cells to Chk1 inhibition was determined. Complete removal of glucose and especially glutamine from the cell culture media decreased the fraction of cells staining positive for γH2AX following Chk1i treatment (Fig. 7A). Titrating in the amount of glucose or glutamine into the cell culture media resulted in a concentration dependent increase in the fraction of γH2AX-positive nuclei following treatment with 1 μM Chk1i that correlated closely with the fraction of actively replicating cells (EdU-positive nuclei) (Fig. 7B). Inhibition of DNA replication through the removal of glucose or glutamine rendered cells refractory to Chk1i induced DNA damage. Chk1i induced DNA damage was restored in glutamine starved cells by the reversal of nutrient starvation. Addition of glutamine to HT29 or U2OS cells starved of glutamine for 24 hours resulted in a restart of DNA replication and an increase in Chk1i induced DNA damage (Fig. 7C). Inhibition of cell growth and division through restriction of glutamine decreased Chk1 kinase activity and rendered cancer cells less sensitive to Chk1 inhibition.

## Discussion

Chk1 inhibitors, either as monotherapy or in combination with cytotoxic chemotherapy, are currently under clinical evaluation in a range of Phase I and II trials. Chk1 inhibitors most effectively enhance the cytotoxicity of anti-metabolite drugs such as pemetrexed, gemcitabine or hydroxyurea *in vitro* and *in vivo*^18^. These drugs generally work by inhibiting enzymes critical for nucleotide or deoxyribonucleotide biosynthesis decreasing the pool of dNTPs available for DNA synthesis. Chk1 inhibition, in combination with antimetabolite chemotherapy, results in the collapse and subsequent cleavage of stalled replication forks, increased DNA double strand breaks and cell death via apoptosis, necrosis, mitotic catastrophe or senescence^22^,^23^,^25^. We therefore hypothesised that inhibiting other metabolic pathways critical for the supply of building blocks necessary to support DNA replication may lead to increased DNA damage and synergy with an inhibitor of Chk1.

A screen of 11 compounds with diverse effects on cellular metabolic pathways identified synergistic activity between the Chk1 inhibitor V158411 and chloroquine or GSK 2837808A. Chloroquine is an anti-malarial drug that also inhibits autophagy through its accumulation in lysosomes and inhibition of lysosomal enzymes. Inhibition of autophagy has previously been demonstrated to enhance proteasomal degradation of Chk1^26^ and Chk1 inhibition with the small molecule inhibitor LY2603618 resulted in increased autophagy^27^. This suggests that inhibition of Chk1 inhibitor-induced autophagy coupled with increased Chk1 protein degradation, thereby reducing the concentration of Chk1i needed to induce DNA damage, underlies the observed synergy between CHL and Chk1i.

GSK 2837808A is a potent inhibitor of lactate dehydrogenase enzymes LDHA and LDHB (IC_50_ 1.9 and 14 nM respectively^28^). In comparison to normal cells, cancer cells derive a large amount of their ATP through the conversion of glucose to lactate in the cytosol (a process termed aerobic glycolysis or the Warburg effect). LDH enzymes reduce the growing pool of pyruvate to lactate thereby regenerating nicotinamide adenine dinucleotide. Inhibition of LDH results in decreased cytosolic glucose processing coupled with increased Krebs cycle activity and mitochondrial processing of cellular pyruvate. Increased ROS and subsequently DNA damage due to the metabolic switch could underlie the observed synergy. However, no combinatorial activity was observed between Chk1i and the LDHA inhibitor oxamic acid. This may reflect differences in the potency and selectivity of the two compounds. Billiard *et al*.^28^ note that at doses of GSK 10 μM and higher (thereby covering the concentrations at which combinatorial activity with Chk1i was observed) mitochondrial effects that are likely not mediated by LDH inhibition were exhibited.

Given the mechanism of action of certain compounds coupled with previously published observations, the lack of combinatorial activity between Chk1i and simvastin or TH-588 was surprising. Simvastin, a commonly prescribed statin, is a potent inhibitor of HMG-CoA (IC_50_ 11.2 nM), the rate limiting enzyme in the endogenous production of cholesterol. Disruption of this pathway with 6-fluoromevalonate, an inhibitor of mevalonate-PP decarboxylase, decreased dNTP pools and induced DNA damage without affecting cellular cholesterol levels^29^. The lack of combinatorial activity between Chk1i and simvastin may be due to the presence of sufficient cholesterol in the cell culture media to compensate for HMG-CoA inhibition. Alternatively, 6-fluoromevalonate may modulate additional enzymes to mevalonate-PP decarboxylase.

TH-588 inhibits MTH1 (IC_50_ 5 nM), an enzyme responsible for the sanitation of oxidised dNTP pools thereby preventing the incorporation of damaged bases into DNA^30^. Inhibition of MTH1 induces DNA damage and selectively inhibits cancer cell survival whilst sparing normal cells. Combinatorial activity between Chk1i and TH was observed in HT29 but not U2OS cells at concentrations of TH greater than or equal to 20 μM. At TH concentrations <20 μM, no combinatorial activity was observed in either cell line. In HT29 cells, TH induced mitotic arrest whilst in U2OS cells, treatment with TH induced cell death as determined by an increase in the sub-G1 population. Replication of 8-oxoG incorporated into DNA results in a mutagenic mismatched base pairing with adenine. OGG1 is the major human repair enzyme of DNA incorporated 8-oxoG whilst hMYH excises the adenine mis-incorporated opposite the 8-oxoG. It is likely that whilst these lesions are extremely mutagenic, they do not result in replication fork arrest, increased replication stress and ATR-Chk1 pathway activation.

Metabolic reprogramming in cancer cells increases the cellular demand for glucose and glutamine to provide the necessary building blocks for biosynthesis. Removal of the cellular supply of glucose or glutamine decreased Chk1 protein levels as well as reducing the amount of active Chk1. This correlated with a dramatic decrease in the fraction of cells undergoing active DNA synthesis without a consequent induction of DNA damage. This strongly suggests that in an unperturbed cell cycle, Chk1 is expressed predominantly in S-phase to deal with the consequences of DNA damage arsing due to endogenous replication stress. Replication stress can arise through numerous mechanisms with different oncogenes triggering replication stress through multiple different mechanisms. Loss of the controls restricting the onset of S-phase results in an unscheduled and uncoordinated replication burst, that is not matched by the supply of components necessary for replication fork progression, resulting in replication fork stalling, fork collapse and the generation of DNA double strand breaks (DSBs).

Withdrawal of glucose or glutamine rendered tumour cells refractory to Chk1i-induced DNA damage and this could be reversed by refeeding the cells with the required nutrients. This provides a rationale for the antagonistic nature of compounds such as 2DG or 6AN that inhibit key metabolic processes resulting in inhibition of DNA replication without DNA damage. These observations have important ramifications for the clinical development of Chk1 inhibitors as monotherapy agents, namely: (i) tumours with a large fraction of actively replicating cells are predicted to be responsive, (ii) schedules and/or inhibitors that maintain Chk1 inhibition over several days (thereby targeting tumour cells as they enter and progress through replication) may provide greater efficacy, and (iii) quiescent or poorly vascularised regions of a tumour will be refractory to therapy.

## Methods

### Cell lines and cell culture

Cell lines were purchased from the American Type Culture Collection (ATCC), established as a low passage cell bank and then routinely passaged in our laboratory for less than 3 months after resuscitation. These were routinely cultured in media containing 10% FCS and 1% penicillin/streptomycin at 37 °C in a normal humidified atmosphere supplemented with 5% CO_2_. Cells were authenticated by STR profiling (LGC Standards, Teddington UK).

### Compounds

Solid stocks were purchased from the indicated suppliers and prepared as concentrated stock solutions in the appropriate solvent: hydroxyurea (100 mM in dH_2_O) from Acros, 2-deoxygluosce (1 M in dH_2_O), oxamate (100 mM in dH_2_O), 6-aminonicotinamide (2.5 mM in DMSO), piperlongumine (20 mM in DMSO), simvastin (20 mM in DMSO), L-buthionine-sulfoxamine (50 mM in dH_2_O) or chloroquine (20 mM in dH_2_O) from Sigma; GSK 2837808A (10 mM in DMSO) and metformin (100 mM in DMEM) from TOCRIS bioscience and TH588 (10 mM in DMSO) from Selleckchem. V158411 and VER-246008 were from Vernalis Research and prepared as 20 mM DMSO stocks. Compounds were serially diluted in the appropriate solvent to 500× or 1000× final concentration then to 5× or 10× final concentration in complete media before addition to cells to yield a 1× final concentration.

### Antibodies

Antibodies against Chk1, pChk1 (S317), pChk2 (T68), pH2AX (S139), pCdc2 (Y15), pHH3 (S10), PCNA, CDT1, MCM2, Geminin and GAPDH were purchased from Cell Signaling Technologies; pChk1 (S296), RPA32 and Cdc6 from Abcam; pRPA32 (S4/S8) from Bethyl Laboratories and pH2AX (S139) (clone JBW301) from Merck Millipore. Antibodies were used at the manufacturer’s recommended dilutions.

### Immunoblotting

Cells were washed once with PBS and lysed in RIPA buffer containing protease and phosphatase inhibitor cocktails (Roche). Protein concentration was determined using a BCA kit (Pierce). Equal amounts of lysate were separated by SDS-PAGE and western blot analysis conducted using the antibodies indicated above. Primary antibodies were detected with HRP-conjugated secondary antibody (Santa Cruz Biotechnology) and detected with Western Lightning (Perkin Elmer) or Immobilon (Millipore) chemiluminescent HRP substrate. Blots were imaged using an LAS 4000 luminescence imager (Fujifilm). Densitometry was determined using Image J software (NIH).

### Single Cell Immunofluorescent Imaging

Following compound treatment, cells were fixed in 3.7% paraformaldehyde in PBS at room temperature for 15 minutes, washed with PBS, blocked with 5% normal goat serum in 0.3% Triton X100 in PBS for 1 hour at room temperature then incubated with primary antibody diluted in antibody dilution buffer (1% BSA, 0.3% Triton X100 in PBS) at 4 °C for 16 hours. Cells were washed with PBS then incubated with an Alexa-labelled secondary antibody (1:500, Life Technologies) and Hoechst 33342 (1 μg/ml) in antibody dilution buffer at room temperature for 60 minutes. Following washing with PBS, cells were imaged with an Operetta high content imaging system (Perkin Elmer) at 10× or 20× magnification and analysed using Harmony software (Perkin Elmer).

### High Content Cell Cycle Analysis

High content cell cycle analysis was conducted essentially as previously described^31^. For DNA only analysis, cells were fixed and permeabilised with 3.7% paraformaldehyde/0.3% Triton X100 in PBS at room temperature for 15 minutes. Cells were washed twice in PBS then stained with Hoechst 33342 (1 μg/ml) in PBS at room temperature for 30 minutes.

For multiparametric cell cycle analysis, cells were labelled with 10 μM EdU for 15 minutes immediately prior to fixation with 3.7% paraformaldehyde in PBS at room temperature for 15 minutes. Cells were washed twice in PBS then twice in 3% BSA in PBS before permeabilisation with 0.5% Triton X100 in PBS for 20 minutes at room temperature. Cells were washed twice with 3% BSA in PBS before incorporated EdU was labelled with an Alexa Click-iT EdU labelling kit (Life Technologies). Following blocking for 30 minutes with 5% normal goat serum in PBS, cells were incubated with an anti-pHH3 (S10) primary antibody diluted in antibody dilution buffer (1% BSA, 0.3% Triton X100 in PBS) at 4 °C for 16 hours. Cells were washed with PBS then incubated with an Alexa-labelled secondary antibody (1:500, Life Technologies) and Hoechst 33342 (1 μg/ml) in antibody dilution buffer at room temperature for 60 minutes. Following washing with PBS, cells were imaged with an Operetta high content imaging system (Perkin Elmer) at 10× magnification and analysed using Harmony software (Perkin Elmer).

### Cell Proliferation Assay

5000 cells per well were seeded in 96-well plates and incubated overnight. Cells were treated with a 10-point titration of compound for 72 hours. The effect on cell proliferation was determined with sulphorhodamine B (SRB) after fixation with 10% trichloroacetic acid and read on a Victor plate reader (Perkin Elmer). GI_50_ values were calculated in Microsoft EXCEL using an XLFit software add-in (ID Business Solutions). For γH2AX and pChk1 (S317), synergy was determined using the model of Bliss Independence where the Combination Index (CI) = E_A_ + E_B_ − E_A_.E_B_/E_AB_. For cytotoxicity assays, Loewe, Bliss and HSA drug synergy scores were calculated using Combenefit software^32^.

### Statistical Analysis

Results were analyzed using a 2-tailed Student’s t-Test tool within the data analysis package provided by Microsoft Excel.

## Additional Information

**How to cite this article**: Massey, A. J. Modification of tumour cell metabolism modulates sensitivity to Chk1 inhibitor-induced DNA damage. *Sci. Rep.* **7**, 40778; doi: 10.1038/srep40778 (2017).

**Publisher's note:** Springer Nature remains neutral with regard to jurisdictional claims in published maps and institutional affiliations.

## Supplementary Material

Supplementary Tables and Figures

## Figures and Tables

**Table 1:** Compounds used in the study and their mechanism of action.

Compound	Abrv	Test Conc (mM)^a^	Target/Mechanism	Refs
V158411	Chk1i		Potent, selective inhibitor of Chk1	22
Hydroxyurea	HU	2.5	Ribonucleoside diphosphate reductase inhibitor	
2-deoxyglucose	2DG	20	Inhibitor of glucose phosphorylation by hexokinase	
Metformin	MET	20	Activator of LKB1/AMPK pathway	33
Oxamic Acid	OX	20	Lactate dehydrogenase A (LDHA) inhibitor	
GSK 2837808A	GSK	0.08	Lactate dehydrogenase (LDHA & LDHB) inhibitor	28
6-aminonicotinamide	6AN	0.05	6-phosphogluconate dehydrogenase inhibitor	34
Simvastin	SIM	0.001	HMG-CoA reductase inhibitor	
Piperlongumine	PIP	0.002	Inducer of ROS	35
TH588	TH	0.02	MTH-1 (NUDT1) inhibitor	30
L-buthionine-sulfoxamine	LBUT	1	Irreversible inhibitor of γ-glutamylcysteine synthetase	
Chloroquine	CHL	0.08	Antimalarial drug. Autophagy inhibitor	
VER-246008	VER	0.08	Pyruvate dehydrogenase kinase (PDHK1-4) inhibitor	36

^a^Test concentrations were selected based on the literature. These were chosen to be clinically relevant where appropriate.
