# Supplementary material for: Modification of tumour cell metabolism modulates sensitivity to Chk1 inhibitor-induced DNA damage
Source: Sci Rep. 2017 Jan 20;7:40778. doi: 10.1038/srep40778 (PMC5247758; doi:10.1038/srep40778)
Supplement: Supplementary Tables and Figures [file srep40778-s1.pdf]

Modification of tumour cell metabolism modulates sensitivity to Chk1 inhibitor-induced DNA damage

Andrew J Massey

Supplementary Information

Supplementary Table S1. Statistical comparisons for figure 1A.

Combination	Single Agent (SA)											
	HU	2DG	MET	OX	GSK	6AN	SIM	PIP	TH	LBUT	CHL	VER
HT29 pH2AX												
SA vs DMSO	***	ns	ns	ns	*	*	ns	**	**	ns	***	***
Combo vs SA	***	***	***	***	***	***	***	***	***	***	***	ns
Combo vs Chk1i	***	***	***	***	*	***	ns	**	**	ns	**	***
HT29 pChk1												
SA vs DMSO	***	*	ns	ns	*	***	ns	ns	**	ns	***	***
Combo vs SA	*	***	***	***	***	ns	***	***	***	***	ns	**
Combo vs Chk1i	**	*	***	***	*	ns	ns	**	***	ns	***	**
U2OS pH2AX												
SA vs DMSO	***	*	*	ns	**	ns	ns	ns	***	ns	ns	ns
Combo vs SA	***	***	**	ns	***	***	***	***	***	***	***	***
Combo vs Chk1i	***	***	***	***	**	***	ns	***	ns	ns	***	*
U2OS pChk1												
SA vs DMSO	***	ns	ns	*	**	ns	ns	*	***	*	ns	**
Combo vs SA	**	*	ns	ns	*	ns	***	**	***	***	***	***
Combo vs Chk1i	***	*	***	***	**	**	ns	**	ns	ns	***	*

Supplementary Table S2. Densitometric analysis of HT29 western blots from figure 1B.

	Percentage Change Relative to DMSO											
	HU	2DG	MET	OX	GSK	6AN	SIM	PIP	TH	LBUT	CHL	VER
pS296	3.2	0.9	2.0	2.1	0.9	0.6	2.0	2.2	0.4	1.6	0.4	0.4
pS317	29.5	3.4	0.6	0.6	1.3	0.7	1.4	1.1	0.3	1.2	6.6	1.9
Chk1	0.5	0.5	0.7	0.6	0.5	0.3	0.5	0.5	0.5	0.7	0.3	0.3
pChk2	3.4	1.5	1.7	1.3	1.1	0.8	1.0	1.0	0.8	1.1	0.7	2.8
pRPA	6.1	0.7	0.7	0.5	0.4	0.4	0.2	0.3	0.3	0.5	0.9	0.5
H2AX	3.9	3.0	2.1	3.0	1.3	1.8	0.9	0.6	3.7	1.4	10.5	6.2

Densitometric analysis was conducted using Image J software. Band intensities were normalised to Chk1 for pS296 and pS317 and to GAPDH for all others, and fold change relative to DMSO calculated.

Supplementary Table S3. Densitometric analysis of U2OS western blots from figure 1B.

	Percentage Change Relative to DMSO											
	HU	2DG	MET	OX	GSK	6AN	SIM	PIP	TH	LBUT	CHL	VER
pS296	0.9	0.2	0.7	0.5	0.1	0.1	0.3	0.7	0.3	0.4	0.5	0.3
pS317	8.8	2.0	2.2	0.6	0.9	0.9	1.2	1.5	1.4	1.9	2.3	1.1
Chk1	1.0	0.8	1.2	1.0	0.9	1.1	1.1	1.1	0.7	1.1	0.9	0.9
pChk2	6.6	1.9	1.7	1.1	1.2	1.1	1.1	0.9	1.0	1.2	0.8	1.3
pRPA	30.8	6.6	1.9	1.5	1.9	1.2	1.0	1.9	2.0	1.5	0.2	1.7
H2AX	1.0	0.8	1.0	0.4	0.3	1.7	3.3	1.7	3.3	1.9	1.4	0.5

Densitometric analysis was conducted using Image J software. Band intensities were normalised to Chk1 for pS296 and pS317 and to GAPDH for all others, and fold change relative to DMSO calculated.

A

HT29

	pH2AX +ve (%)				
V411	GSK 2837808A (μM)				
(μM)	80	40	20	10	5
1.0	1.04	0.98	1.00	0.99	1.00
0.3	0.51	0.75	0.87	1.00	0.91
0.1	0.80	1.25	1.47	1.45	1.45

	pH2AX +ve (%)				
V411	CHL (μM)				
(μM)	80	40	20	10	5
1.0	0.89	0.88	0.82	0.83	0.84
0.3	0.18	0.28	0.27	0.29	0.41
0.1	0.18	0.42	0.58	0.63	0.75

	pChk1 +ve (%)				
V411	GSK 2837808A (μM)				
(μM)	80	40	20	10	5
1.0	1.08	0.98	1.02	1.00	1.03
0.3	0.65	1.02	1.01	1.19	1.06
0.1	2.65	1.78	2.28	1.81	1.85

	pChk1 +ve (%)				
V411	CHL (μM)				
(μM)	80	40	20	10	5
1.0	1.11	0.82	0.69	0.73	0.76
0.3	0.81	0.43	0.20	0.29	0.55
0.1	1.00	0.96	1.02	1.70	1.59

U2OS

	pH2AX +ve (%)				
V411	GSK 2837808A (μM)				
(μM)	80	40	20	10	5
1.0	1.53	1.08	0.92	0.88	0.91
0.3	0.20	0.09	0.16	0.33	0.62
0.1	0.45	0.28	0.50	0.87	1.17

	pH2AX +ve (%)				
V411	CHL (μM)				
(μM)	80	40	20	10	5
1.0	0.94	0.94	0.97	0.98	0.97
0.3	0.12	0.19	0.35	0.56	0.80
0.1	0.05	0.33	0.96	1.28	1.03

	pChk1 +ve (%)				
V411	GSK 2837808A (μM)				
(μM)	80	40	20	10	5
1.0	1.69	1.09	0.88	0.86	0.91
0.3	0.41	0.16	0.30	0.51	0.76
0.1	1.21	0.89	1.38	1.28	1.59

	pChk1 +ve (%)				
V411	CHL (μM)				
(μM)	80	40	20	10	5
1.0	0.97	0.94	0.97	1.01	0.96
0.3	0.16	0.31	0.52	0.75	0.98
0.1	0.22	0.94	1.39	1.31	1.34

B

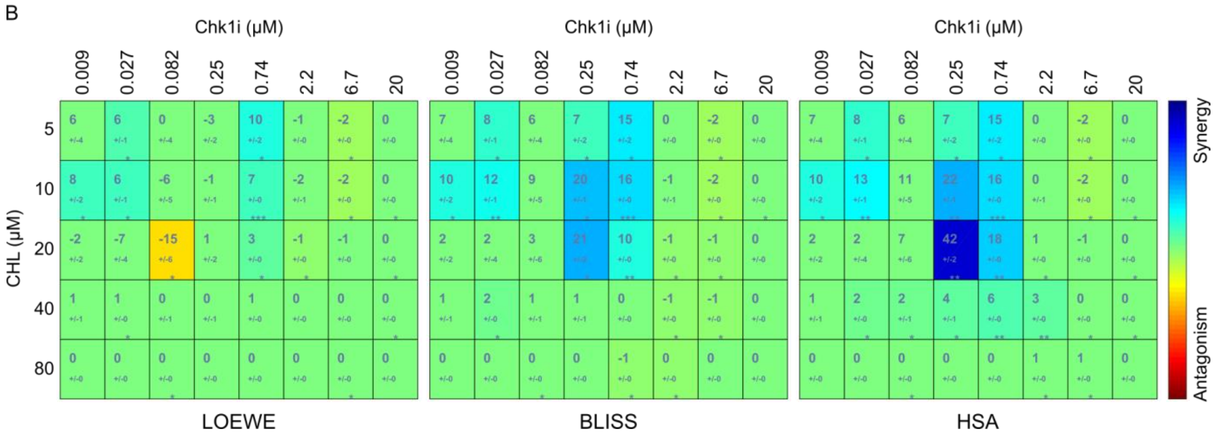

Supplementary Figure S1. Related to figure 2.
(A) Combination Index (CI) scores were calculated from the data in figure 2A using the model of Bliss Independence. Combinations where the CI < 0.75 (indicating synergy) are highlighted in red. (B) Synergy (Loewe, Bliss and HSA) for the combination of CHL with Chk1i in HT29 cells was calculated using Combenefit software.

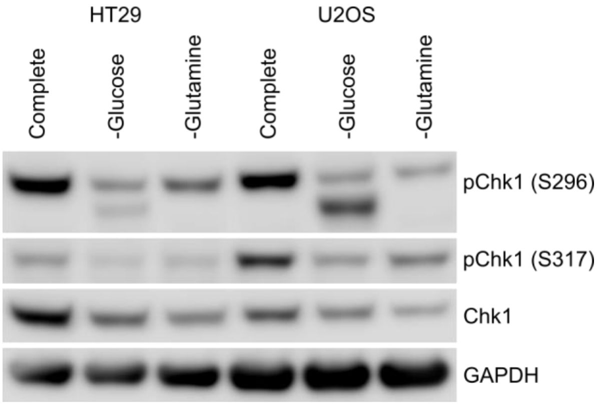

Supplementary Figure S2. Related to figure 6. Repeat western blot of figure 6C.
